# Supplementary material for: Effects of Irrigation and Fertilization on the Morphophysiological Traits of Populus sibirica Hort. Ex Tausch and Ulmus pumila L. in the Semiarid Steppe Region of Mongolia
Source: Plants (Basel). 2021 Nov 8;10(11):2407. doi: 10.3390/plants10112407 (PMC8620301; doi:10.3390/plants10112407)
Supplement: Supplementary file 1 [file plants-10-02407-s001.zip › plants-1434210-supplementary.pdf]

Table S1. P-values of Two-way ANOVA for treatment effects on growth and morpho-physiological traits of *Populus sibirica* and *Ulmus pumila*

Species	year	Variables	Treatments	DF	MS	F Value	Pr>F
Populus sibirica	2018	SLA	irrigation	3	915.716068	28.40	<0.0001
			fertilization	2	300.876691	9.33	0.0002
			Irrigation x fertilization	4	16.397573	0.51	0.7295
		LA	irrigation	3	1128.536640	10.24	<0.0001
			fertilization	2	921.606120	8.36	0.0004
			Irrigation x fertilization	4	155.595581	1.41	0.2350
		LB	irrigation	3	0.07169171	5.77	0.0011
			fertilization	2	0.06597823	5.31	0.0063
			Irrigation x fertilization	4	0.01679479	1.35	0.2554
		Chlorophyll content total	irrigation	3	39.4152069	13.23	<0.0001
			fertilization	2	38.0227070	12.76	<0.0001
			Irrigation x fertilization	4	50.6819682	17.01	<0.0001
		Chlorophyll a/b ratio	irrigation	3	9.92541782	2.45	0.0701
			fertilization	2	7.76034473	1.92	0.1544
			Irrigation x fertilization	4	6.96214996	1.72	0.1548
		Water potential (13:00)	irrigation	3	0.03416275	3.06	0.0914
	2019	SLA	irrigation	3	240.7631521	3.35	0.0218
			fertilization	2	149.5014584	2.08	0.1299
			Irrigation x fertilization	4	61.1093985	0.85	0.4963
		LA	irrigation	3	0.14923105	5.74	0.0011
			fertilization	2	0.09053664	3.48	0.0343
			Irrigation x fertilization	4	0.13100318	5.04	0.0009
		LB	irrigation	3	1829.499087	5.80	0.0010
			fertilization	2	1218.928202	3.86	0.0239
			Irrigation x fertilization	4	1189.296191	3.77	0.0065
		Chlorophyll content total	irrigation	3	137.4831006	37.75	<0.0001
			fertilization	2	47.4115287	13.02	<0.0001
			Irrigation x fertilization	4	51.5728782	14.16	<0.0001
		Chlorophyll a/b ratio	irrigation	3	0.14451951	3.98	0.0107
			fertilization	2	0.07993435	2.20	0.1172
			Irrigation x fertilization	4	0.53869764	14.84	<0.0001
		Water potential (13:00)	irrigation	3	0.03416275	3.06	0.0914
Ulmus pumila	2018	SLA	irrigation	3	554.802018	12.79	<0.0001
			fertilization	2	815.337819	18.79	<0.0001
			Irrigation x fertilization	4	64.052395	1.45	0.2234
		LA	irrigation	3	89.8025298	8.07	<0.0001
			fertilization	2	90.5970466	8.14	0.0005
			Irrigation x fertilization	4	4.6425834	0.38	0.8197
		LB	irrigation	3	0.00772819	6.11	0.0007
			fertilization	2	0.00704955	5.57	0.0050
			Irrigation x fertilization	4	0.00031831	0.25	0.9081
		Chlorophyll content total	irrigation	3	72.9263154	11.45	<0.0001
			fertilization	2	15.3498171	2.41	0.0963
			Irrigation x fertilization	4	38.1000451	5.98	0.0003
		Chlorophyll a/b ratio	irrigation	3	2.02889914	0.96	0.4154
			fertilization	2	5.60755241	2.66	0.0764

			Irrigation x fertilization	4	8.65418861	4.10	0.0045
		Water potential (13:00)	irrigation	3	0.02335489	0.90	0.4817
	2019	SLA	irrigation	3	835.150788	8.01	<0.0001
			fertilization	2	1849.083921	17.74	<0.0001
			Irrigation x fertilization	4	1764.802836	16.93	<0.0001
		LA	irrigation	3	0.00178832	1.41	0.2446
			fertilization	2	0.00617236	4.86	0.0095
			Irrigation x fertilization	4	0.00988866	7.78	<0.0001
		LB	irrigation	3	59.6299359	7.02	0.0002
			fertilization	2	159.2996141	18.76	<0.0001
			Irrigation x fertilization	4	22.2091465	2.62	0.0390
		Chlorophyll content total	irrigation	3	107.2576607	17.39	<0.0001
			fertilization	2	56.9739893	9.24	0.0002
			Irrigation x fertilization	4	2.8817998	0.47	0.7596
		Chlorophyll a/b ratio	irrigation	3	1.58999099	8.54	<0.0001
			fertilization	2	0.02500083	0.13	0.8746
			Irrigation x fertilization	4	1.84875884	9.93	<0.0001
		Water potential (13:00)	irrigation	3	0.10895344	3.70	0.0616
